# Survey dataset on key drivers of tax evasion

**DOI:** 10.1016/j.dib.2026.112755

**Published:** 2026-04-07

**Authors:** Salem A. Al-Jundi, Reshmi Augustine

**Affiliations:** a College of Administration and Economics, Al-Kunooze University, Basra, Iraq; b Department of Qualification Development, Rabdan Academy, Abu Dhabi, United Arab Emirates

**Keywords:** Deviant culture, Law enforcement, Administrative corruption, Taxpayers, Baghdad, Iraq

## Abstract

Tax evasion remains a challenge for public revenue systems, and is influenced by institutional and cultural factors. This article provides a dataset to empirically evaluate and operationalize the interrelationships among these factors in the context of Iraq. Although previous research has investigated deviant culture, weak law enforcement, and administrative corruption on an individual basis, few datasets document these factors within a unified measurement structure. An online questionnaire was employed to gather data from 459 taxpayers in Baghdad, Iraq, from September–November 2021. The dataset operationalizes four constructs: tax evasion (illegal non-payment or underpayment of taxes through underreporting income, inflating deductions, or concealing financial sources), deviant culture (social norms and behavioral patterns that eschew accepted moral standards), weak law enforcement (limitations in the state’s capacity or willingness to enforce laws and regulations), and administrative corruption (misuse of public office for private benefit, commonly reflected through, e.g., bribery and favoritism). Data were collected using an online questionnaire administered via Google Forms and developed using reflective measurement models, with each construct using multiple indicators adapted from sources validated in the literature. Responses were rated on a 7-point Likert-type scale. The instrument was translated into Arabic, subjected to cultural review, and pilot tested to ensure clarity and contextual suitability. In addition to the indicators, the dataset included five demographic variables that described respondent characteristics. The collection consisted of anonymized raw responses, coding schemes, construct indicators, and metadata files, which included variable definitions, response coding, and item references. This dataset offers a comprehensive measurement framework for analyzing tax-related behaviors in developing contexts. It encourages the replication of measurement models, cross-country comparative research, and secondary analyses of taxpayer perceptions of governance, culture, and enforcement. Transparency, reusability, and evidence-based policymaking in tax governance are facilitated by the dataset’s public availability and accompanying documentation on Mendeley Data.

Specifications Table

**Table:** 

Subject	Social Sciences
Specific subject area	Perceptions of taxpayers on the pervasiveness of tax evasion and its key drivers
Type of data	Table (.xlsx format), figure Raw, processed Supporting materials (codes and text version of survey)
Data collection	Based on a review of the literature, key drivers of tax evasion include deviant culture, weak law enforcement, and administrative corruption. These constructs were measured using reflective measurement models and a 7-point Likert scale. The survey, conducted in Baghdad, Iraq, comprised two sections; the first assessed these constructs through corresponding observed variables and the second covered taxpayer demographics. It was distributed via Google Forms, and data collection spanned three months (Sept.–Nov. 2021), yielding 459 complete and usable responses.
Data source location	City: Baghdad Country: Iraq Latitude: 33° 19′ 08.3″ N Longitude: 44° 21′ 55.9″ E
Data accessibility	Repository name: Mendeley Data Data identification number: https://doi.org/10.17632/G75TVDVRZG.1 Direct URL to data: https://data.mendeley.com/datasets/g75tvdvrzg/1 S. Al-Jundi, Dataset on tax evasion in Baghdad, Iraq, Mendeley Data V1 (2025). https://doi.org/10.17632/G75TVDVRZG.1.
Related research article	S. A. Al-Jundi, R. Augustine, H. A. Al-Janabi, The effect of deviant culture on tax evasion through law enforcement and administrative corruption, Int. J. Sociol. Soc. Pol. 45 (13/14) (2025) 56–72. https://doi.org/10.1108/IJSSP-03-2025–0173 [1]

## 1 Value of the Data

• These data offer structured and measurable insights into the impact of administrative corruption, ineffective law enforcement, and deviant culture on tax evasion behaviors. The dataset enables researchers to conduct an empirical exploration of these dynamics by implementing each construct using validated indicators, with particular emphasis on developing contexts such as Iraq [2].
• This dataset is suitable for reusing comparative analyses of tax compliance. To examine differences in institutional trust, enforcement practices, and cultural norms across governance systems, it is feasible to utilize similar reflective measurement models and survey structures in other national or regional contexts.
• This dataset can be utilized by policymakers and economists to assess public perceptions of institutional performance and to assist in the development of tax compliance strategies. The data can be used to identify areas in which revenue collection efforts may be undermined by enforcement gaps or trust deficits.
• This dataset serves as a valuable resource for decision-makers and economic analysts to evaluate public views on institutional effectiveness and formulate strategies for tax compliance. This information can be utilized to identify regions in which revenue collection efforts might be compromised due to enforcement shortcomings or lack of trust.
• The methodological reference provided by the survey’s structure, consisting of a 7-point Likert scale and reflective indicators, was adaptable. This could be modified to guarantee consistent and rigorous data collection in studies of taxpayer in comparable socioeconomic environments.

## 2 Background

The data presented in this article were compiled in the context of Iraq’s ongoing institutional and governance challenges, which became particularly visible following the 2022 “Tax Deposit Theft” scandal, commonly referred to as the “Theft of the Century.” The incident involved multibillion-dollar losses and highlighted weaknesses in administrative controls, regulatory enforcement, and public accountability mechanisms [3]. These concerns align with broader governance indicators, including Iraq’s ranking of 154 out of 180 countries in the 2023 Corruption Perceptions Index [4].

Data were generated using a structured survey instrument developed to capture perceptions of tax compliance behavior in this setting. The survey evaluated administrative corruption, weak law enforcement, and deviant cultural norms. These dimensions were defined as latent constructs and measured through reflective indicators that were modified from the existing empirical literature. Information regarding demographic traits typically linked to taxpayer behavior was also collected to support descriptive and comparative analyses. The current data article offers methodological insights into the collection and quantification of data on hidden socioeconomic issues, providing a valuable foundation for future research and policy reforms aimed at tax compliance and governance.

## 3 Data Description

Tax evasion remains a persistent challenge for public revenue systems, and is influenced by institutional and cultural factors. The dataset presented in this article was compiled to capture taxpayers’ perspectives on deviant culture, weak law enforcement, and administrative corruption. These factors were regarded as interrelated dimensions within the dataset, and recorded to capture their coexistence within the surveyed context.

Prior research has documented cultural, legal, and institutional conditions linked to tax evasion [5,6]. In this context, deviant cultural norms, weak law enforcement, and administrative corruption have frequently been examined as related aspects [7,8]. This dataset treats these factors as coexisting conditions and records their mutual presence within the observed setting.

For clarity, the dataset was organized into four core variables. Tax evasion is defined as the illegal avoidance of tax obligations through practices such as hiding income, exaggerating deductions, or inaccurately reporting financial information [9,10]. Deviant culture refers to social norms and behavioral patterns that deviate from established moral standards, where unethical practices, such as tax evasion, may become normalized [11,12]. Weak law enforcement indicates limitations in the government’s ability or readiness to enforce laws and regulations, which may allow noncompliant behavior to continue [13,14]. Administrative corruption is the use of public office for personal gain, and often shows up in the form of bribery, favoritism, or abuse of power [15,16].

Across related works, deviant culture, weak law enforcement, and administrative corruption have commonly been discussed as interconnected, rather than isolated, factors. In the dataset presented here, these dimensions were recorded concurrently to capture how perceptions related to cultural norms and institutional environments coexist within the observed setting. This structure allows the dataset to document the concurrent reporting of these conditions without attributing explanatory or causal roles.

Prior studies have investigated deviant culture, weak law enforcement, and administrative corruption as separate conditions linked to tax evasion [5,8]. This dataset records these dimensions to document joint reporting within a single empirical setting. The dataset also captures perceptions of institutional and cultural issues within a non-Western context, reflecting the taxpayer responses gathered in Baghdad, Iraq. This framework enables the dataset to represent the simultaneous reporting of various conditions within the studied context without specifying interactional or mediating relationships.

The dataset presented in this article was compiled to document taxpayer perceptions related to deviant culture, weak law enforcement, administrative corruption, and tax evasion within a single empirical setting. As these constructs are not directly observable, the dataset employs reflective measurement models in which each construct is represented by multiple observed indicators recorded in the survey instrument.

**Fig. 1:**
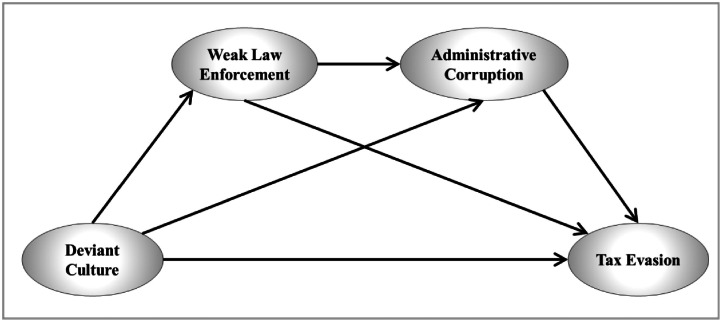
Conceptual model of tax evasion.

In addition, the dataset is linked to our published research, which examines the relationships among deviant culture, weak law enforcement, administrative corruption, and tax evasion [1]. Fig. 1 illustrates how the constructs are conceptually positioned relative to one another, and serves to support understanding of the survey design and variable grouping.

We conducted a survey of 459 individual taxpayers in Baghdad, Iraq’s capital. Within this setting, Baghdad represents an urban environment characterized by economic and demographic diversity alongside documented institutional challenges. Accordingly, the dataset records taxpayers’ perceptions within this specific administrative and social context, providing structured survey data that are relevant to tax evasion and related institutional conditions. The dataset does not include any personal information.

The survey captured four constructs: deviant culture, weak law enforcement, administrative corruption, and tax evasion. As these constructs are not directly observable, they were measured using multiple observed indicators. Responses to all survey items were recorded on a 7-point Likert-type scale ranging from 1 (strongly disagree) to 7 (strongly agree). The indicators were modified from prior empirical research and are included in the accompanying codebook. No modifications beyond numerical coding were implemented throughout the data collection period.

We utilized reflective measurement models to record the constructs within this dataset, as they signify abstract psychological and institutional attributes that cannot be directly observed. In this approach each construct is represented by multiple observed indicators, which are expected to vary and reflect a shared underlying concept. Accordingly, the indicators were developed to provide conceptual alignment and interchangeability. This ensures that variations in the underlying construct are reliably represented across associated measures. This structure also allows researchers to analyze, remove, or replace specific indicators during measurement evaluation without compromising the construct’s conceptual integrity, provided that reliability and validity are maintained.

The distributional properties of the observed variables in the dataset were described using measures of central tendency, such as the mean, median, and mode. These measures typically exhibit comparable values in distributions that approximate normality. However, when distributions are skewed, divergence among the measures is anticipated. In the dataset presented here, most observed variables demonstrated negative skewness, which is defined by distributions that are skewed to the right with longer tails on the left. Under these conditions, the mean was influenced by the lower response values and may not accurately represent the central tendency. This mode may also reflect extreme response categories. Accordingly, the median was reported as a more stable measure of the central tendency to describe the distribution of responses within the dataset.

**Fig. 2:**
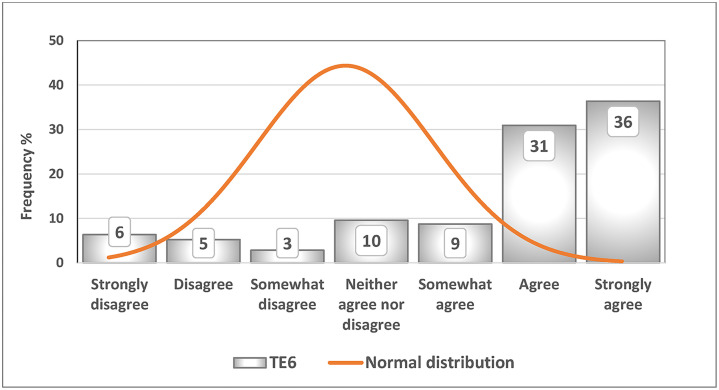
Histogram of TE6 (“People tend to pay bribes to decrease taxes paid”).

Fig. 2 presents the tax evasion indicator TE6 (“People tend to pay bribes to decrease taxes paid”). Among the respondents, 36 % strongly agreed, 31 % agreed, and 9 % somewhat agreed with this statement. Descriptive statistics indicate a mean value of 5.5, which is lower than the median value of 6, whereas the median is lower than the mode value of 7. The order of the mean, median, and mode reflects a non-normal distribution.

**Fig. 3:**
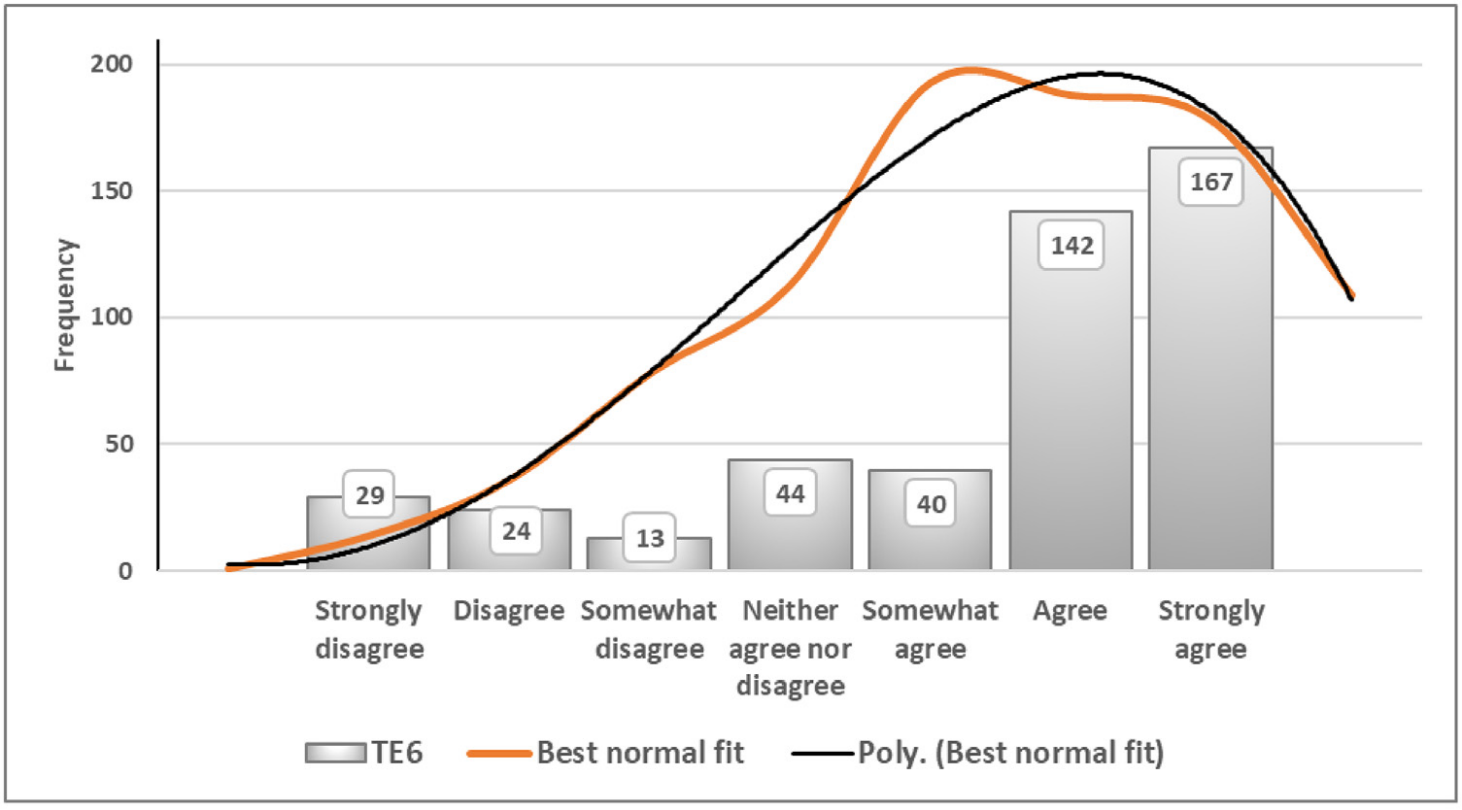
Best normal fit curve of TE6 (People tend to pay bribes to decrease taxes paid).

Fig. 3 further illustrates the distributional pattern of this indicator, showing negative skewness with the distribution shifted to the right and a longer tail on the left. Under these conditions, a median value of 6 provides a more stable measure of central tendency. Overall, 76 % of the 459 respondents reported agreement with the statement.

The tax evasion construct in the dataset was operationalized using measurement items adapted from the scales developed by Khalil and Sidani [9] and Lahiri and Ali [10]. This construct is represented by six observed indicators that document respondents’ acceptance of tax evasion, engagement in ethically questionable tax-related practices, perceptions of taxation as corruptible and exploitative, dissatisfaction with high tax rates and willingness to reduce taxes through bribery.

Table 1 summarizes the descriptive properties of the six tax evasion indicators. The mean values are centered around 5 (*somewhat agree*), whereas the mode is consistently observed at 7 (*strongly agree*). The median value is 6 (*agree*) for most indicators, with one indicator reporting a median of 5. Across all variables, the distributions exhibit negative skewness, with a higher concentration of responses in the upper agreement categories and fewer responses at the lower end of the scale, as observed during the data collection period from September to November 2021.

**Table 1:** Measurement properties of tax evasion.

Code	Item	Mean	Median	Standard Deviation	Skewness	Outer Loading
TE1	People consider tax evasion acceptable.	4.904	6	1.826	−0.793	0.791
TE2	Most people do not consider tax evasion to be immoral.	4.965	6	1.787	−0.821	0.81
TE3	People evade taxes because corruption taints the tax and fee collection departments.	5.418	6	1.762	−1.13	0.884
TE4	People tend to evade taxes since much of the money collected does not seem to benefit them.	5.309	6	1.769	−1.075	0.877
TE5	People tend to evade taxes since the tax rates are too high.	4.88	5	1.85	−0.744	0.685
TE6	People tend to pay bribes to decrease taxes paid.	5.475	6	1.797	−1.256	0.869

*Note:* 7-point Likert scale from 1 (*strongly disagree)* to 7 (*strongly agree)*.

The final column in Table 1 reports the outer loadings for the six tax evasion indicators. Outer loadings exhibit the relationship between an observable indicator and its corresponding construct. In measurement assessment, loadings above 0.7 are commonly treated as acceptable, values below 0.4 are typically excluded, and values between 0.4 and 0.7 may be retained when overall construct validity is supported [1]. All six indicators (TE1–TE6) record outer loading values that are within the acceptable ranges reported in Table 1.

The deviant culture construct in the dataset was operationalized using measurement items adapted primarily from the empirical work of Wahlund [12] and Al-Jundi [17]. These indicators document respondents’ attitudes toward corruption, tolerance of illegal activities, perceived influence of religion on behavior, expressions of greed, and perceptions related to taxation and tax evasion.

The distribution of responses for the deviant culture indicators follows a pattern similar to that observed for the tax evasion indicators. As shown in Table 2, the median value for most items is 6 (*agree*), indicating higher response frequencies in the upper agreement categories. The mode is frequently observed at 7 (*strongly agree*). The response distributions do not approximate normality and instead exhibit negative skewness, reflecting a concentration of responses toward higher agreement levels.

**Table 2:** Measurement properties of deviant culture.

Code	Item	Mean	Median	Standard Deviation	Skewness	Outer Loading
DC1	People’s reactions to corruption are weak.	4.791	6	2.135	−0.672	0.689
DC2	Many people break laws—for instance, traffic and municipal laws.	5.712	6	1.712	−1.597	0.875
DC3	Religious deterrence is no longer an effective factor in preventing public officials from practicing corruption.	5.782	6	1.684	−1.603	0.875
DC4	Corrupt people are characterized by greed.	6.063	7	1.601	−2.035	0.876
DC5	People have negative attitudes toward taxes.	5.536	6	1.61	−1.428	0.843
DC6	People have negative attitudes toward tax evasion.	4.865	6	1.891	−0.784	0.638

*Note:* 7-point Likert scale from 1 (*strongly disagree*) to 7 (*strongly agree*).

With respect to indicator loadings, most deviant culture indicators record values above the 0.7 reference level, as reported in Table 2. Two indicators (DC1 and DC2) show comparatively lower loading values but remain within acceptable ranges, because the overall reliability and validity of the construct are maintained. Accordingly, all six indicators (DC1–DC6) are documented in the dataset to represent the deviant culture construct.

The weak law enforcement construct is represented in the dataset using items adapted from the work of Anders et al. [13] and Swanepoel and Meiring [14], respectively. These indicators capture respondents’ perceptions of related investigative effectiveness, penalty severity and complexity, sentencing leniency, delays in detection and prosecution, and broader societal attitudes toward crime.

As shown in Table 3, the median value for all indicators is 6 (*agree*), the mean values are above 5 (*somewhat agree*), and the mode is consistently observed at 7 (*strongly agree*). The response distributions exhibit negative skewness, with a higher concentration of responses in the upper agreement categories and fewer responses at lower scale values. Together, these statistics summarize the distribution of responses recorded on a 7-point Likert-type scale for each indicator included in the dataset.

**Table 3:** Measurement properties of weak law enforcement.

Code	Item	Mean	Median	Standard Deviation	Skewness	Outer Loading
LE1	Few corruption cases by public officials are detected or investigated.	5.575	6	1.873	−1.293	0.821
LE2	Most corruption cases in courts do not result in punishments.	5.281	6	1.864	−1.048	0.862
LE3	If corrupt officials receive a punishment, it is considered lenient.	5.303	6	1.8	−1.078	0.847
LE4	A case of corruption is usually detected years after it was committed.	5.623	6	1.827	−1.398	0.912
LE5	If a corruption case is detected and investigated, prosecution and judgment take more than two years.	5.416	6	1.799	−1.173	0.907
LE6	People are reluctant to speak about fraud, corruption, or tax evasion, or to report such crimes.	5.035	6	1.952	−0.881	0.728

*Note:* 7-point Likert scale from 1 (*strongly disagree*) to 7 (*strongly agree*).

The outer loading values for all weak law enforcement indicators are listed in Table 3. Each indicator recorded a load above the 0.7 reference level, and no indicator fell below this threshold. These values describe the measurement properties of the indicators included in the dataset.

The administrative corruption construct is represented in the dataset using six observed indicators adapted from prior empirical studies by Al-Jundi et al. [15] and Peerthum and Luckho [16] respectively. These indicators address perceptions related to the prevalence of corruption, benefits obtained by public officials, the presence of unqualified officers, corruption scandals involving officials, bribery practices, and issues associated with government contracts. Item wording, variable coding, and construct assignments are documented in the accompanying codebook [2].

As shown in Table 4, the median response for most administrative corruption indicators is 7 (*strongly agree*), while the median response for the two indicators is 6 (*agree*). The response distributions exhibit negative skewness, with a higher concentration of responses in the upper agreement categories and fewer responses at the lower scale values.

**Table 4:** Measurement properties of administrative corruption.

Code	Item	Mean	Median	Standard Deviation	Skewness	Outer Loading
AC1	Corruption is pervasive in this country.	6.048	7	1.628	−1.903	0.922
AC2	Some public officers serve their own interests rather than those of the public.	5.789	6	1.691	−1.648	0.916
AC3	Most public officers, who are appointed based on their political affinities, are incompetent.	5.837	6	1.623	−1.668	0.93
AC4	Scandals involving public officials indicate an increase in the incidence of corruption.	5.956	7	1.623	−1.797	0.922
AC5	Bribes are commonly used to get quick service in some public organizations.	5.889	7	1.656	−1.672	0.938
AC6	Contractors often need to bribe public officials in order to obtain government contracts.	5.819	7	1.73	−1.634	0.929

*Note:* 7-point Likert scale from 1 (*strongly disagree*) to 7 (*strongly agree*).

The outer loading values of the administrative corruption indicators are presented in Table 4. All indicators record loading values above the 0.7 reference level, and no indicator falls below this threshold. These values describe the measurement properties of the indicators included in the dataset.

We subsequently assessed internal consistency reliability by calculating Cronbach’s alpha (α) and composite reliability (ρC) for each construct. As a general guideline, α, ρC, and ρA should each exceed 0.70. As reported in Table 5, all constructs satisfied these thresholds, with α, ρC, and ρA values above 0.70, and ρA falling between α and ρC for every latent construct. These results confirm adequate internal consistency reliability.

**Table 5:** Validity and reliability estimate of the latent constructs.

Latent constructs	Cronbach’s alpha (α)	rho_A *(*ρ_A_*)*	Composite reliability *(*ρ_C_*)*	Average variance extracted
Administrative corruption	0.967	0.967	0.973	0.858
Deviant culture	0.889	0.909	0.916	0.649
Tax evasion	0.903	0.915	0.926	0.676
Weak law enforcement	0.921	0.926	0.939	0.719

Finally, convergent validity was evaluated using the average variance extracted (AVE). A construct is considered to demonstrate sufficient convergent validity when it explains at least 50 % of the variance in its indicators, meaning that AVE should be ≥ 0.50. As shown in Table 5, the AVE values for all constructs surpassed this threshold, thereby confirming convergent validity.

The numerical results presented in all tables and figures were derived using SmartPLS (v. 3.3.5), recognized as the primary software for PLS-SEM.

## 4 Experimental Design, Materials, and Methods

The dataset was generated to document measurement models related to tax evasion and associated institutional and cultural conditions. Fig. 1 presents the conceptual structure used to organize the survey constructs included in the dataset. The related research article [1] utilized this conceptual structure for hypothesis testing, whereas the present study focuses on data collection and documentation.

We developed and conducted a structured survey to gather responses that reflected taxpayer perceptions in Baghdad, Iraq. Baghdad was chosen as the data collection site because of its status as a significant urban center with a diverse and large taxpayer population, as well as established administrative entities that are relevant to taxation. Moreover, conducting data collection within a single geographical context ensured that respondents’ institutional and regulatory exposure was consistent.

During the data collection period, Iraq's public services and administrative systems operated under conditions of constrained funding, aging infrastructure, and documented instances of administrative corruption. Despite substantial financial and military assistance from the United States, official reports have identified discrepancies within public sector payroll systems, including unverified personnel registrations within the Ministry of Defense [18]. These conditions form part of the broader administrative context in which taxpayers’ perceptions were recorded in the dataset.

**Fig. 4:**
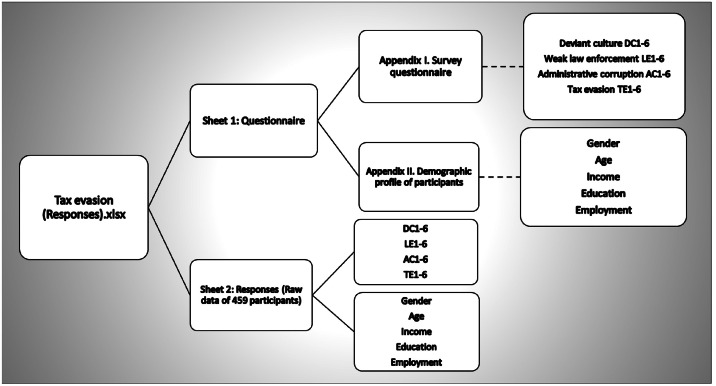
“Tax evasion (responses)” dataset file hosted at Mendeley data.

The questionnaire was divided into two sections. The dataset’s construct indicators, as defined in Appendix I, were the primary focus of the initial section. The demographic characteristics of the respondents were detailed in the second segment, which comprises five items, as specified in Appendix II. To ensure cultural and linguistic appropriateness, the questionnaire was translated into Arabic, the primary language spoken in Baghdad. The translated version was reviewed by five academics from the Middle Technical University in Baghdad to assess clarity and contextual appropriateness. In addition, a pilot exercise was conducted with 30 university students to check item comprehension. Based on this feedback, minor revisions were made to the questionnaire. The final wording of all items is documented in Appendices I and II of the deposited datasets [2]. Fig. 4 illustrates the two sheets included in the datasets. The first sheet contained both sections of the survey questionnaire, whereas the second sheet contained the raw response data corresponding to the indicators summarized in Table 1, Table 2, Table 3, Table 4.

The survey questionnaire was uploaded to Google Forms, and no item could be skipped in order to minimize missing responses. A survey hyperlink was distributed electronically through multiple channels, including e-mail, WhatsApp, Facebook, and other social media platforms. The initial distribution took place among students, administrative staff, and faculty members at Middle Technical University. Participants were subsequently encouraged to share survey links within their personal networks. Data were collected over a three-month period from September to November 2021. At the end of this period, 459 complete and usable responses were obtained and included in the final dataset. The full questionnaire, including construct indicators and demographic items, together with response coding and anonymized raw data, was deposited in Mendeley Data and **is** available in Al-Jundi [2].

A nonprobability sampling approach was used due to the absence of a defined sampling frame for the target population. As outlined by Krejcie and Morgan [19], a sample size of 384 responses corresponds to a 95 % confidence level with *a* ± 5 % margin of error for large populations. Accordingly, the 459 valid responses collected in this study are considered sufficient for evaluating the conceptual model (Fig. 1), while acknowledging the limitations discussed later.

The demographic composition of the sample is summarized using Table 6. The gender distribution of respondents comprised 68 % men and 32 % women. According to population statistics for Baghdad in 2022, the city’s population of approximately 9 million is roughly balanced between men and women [20]. Table 6 presents the age distribution of participants, with 33 % of respondents in their 20 s, 27 % in their 30 s, 12 % in their 40 s and 27 % aged 50 years or above. These figures show the age structure of the surveyed respondents relative to the broader urban population of Baghdad [20].

**Table 6:** Demographic profile of participants.

Characteristics	Classifications	Frequency	Percent
Gender	Female	148	32
	Male	311	68
Age	20 years or younger	9	2
	21–30 years old	152	33
	31–40 years old	122	27
	41–50 years old	54	12
	51 years old or older	122	27
Household monthly income	Less than IQD 500,000	68	15
	IQD 500,000–1000,000	73	16
	IQD 1000,001–1500,000	120	26
	IQD 1500,001–2000,000	63	14
	Above IQD 2000,000	135	29
Educational attainment	Less than secondary school certificate	3	1
	Secondary school certificate	12	3
	A student at college or university	100	22
	Diploma or bachelor’s degree	148	32
	Postgraduate degree or higher	196	43
Employment status	Employed in the government sector	212	46
	Employed in the private sector	99	22
	Self-employed	51	11
	Homemaker	18	4
	Student	54	12
	Others	25	5

Monthly income levels varied, with 15 % of respondents reporting earnings below 0.5 million Iraqi dinars (IQD; approximately 333 USD), 16 % reporting earnings below 1.0 million IQD, 26 % reporting earnings between 1.0 and 1.5 million IQD, 14 % between 1.5 and 2.0 million IQD, and 29 % above 2 million IQD.

The educational attainment of respondents is also measured. The sample included 22 % university or college students, 32 % respondents with a diploma or bachelor’s degree, and 43 % respondents with postgraduate qualifications. A smaller proportion of respondents (3 %) reported secondary school education or lower.

Among the participants, 46 % reported employment in the public sector and 22 % in the private sector, 12 % were students, and 11 % were self-employed. This reflects the centrality of public sector employment in Baghdad’s economy while also capturing the perspectives of private sector employees, students, and independent workers.

Overall, the demographic variables collected in the dataset provide an overview of respondents’ age, income, and employment status. As the dataset documents participation across multiple employment categories and income levels, it provides structured information on the characteristics of surveyed taxpayers in urban Baghdad.

## Limitations

Several limitations related to the data scope and collection should be noted. First, the data collection was limited to taxpayers residing in Baghdad. Although the sample included respondents from varied demographic backgrounds within the city, the dataset may not reflect the perceptions of other Iraqi regions with different administrative, economic, or cultural conditions. Accordingly, the dataset should be interpreted as context-specific, rather than nationally representative. Second, all variables were determined based on respondents’ self-reported survey responses. Despite the anonymous format of the questionnaire, responses may have been influenced by social desirability or respondent hesitation due to the sensitive nature of tax evasion and corruption-related topics. Third, the dataset was collected using a cross-sectional design during a single period from September to November 2021. Consequently, the data captured perceptions at that point in time and did not account for potential temporal variations. Finally, the dataset relies on a predefined set of observed indicators for each construct. Although these indicators were selected based on prior empirical studies, they may not capture all possible dimensions of complex social and institutional phenomena across different governance or cultural settings.

## Ethics Statement

The authors have read and followed the ethical requirements for publication in Data in Brief and confirmed that the current work does not involve human subjects, animal experiments, or any dataa collected from social media platforms. Moreover, they have agreed to authorship, read and approved the manuscript, as well as given consent for submission and subsequent publication of the manuscript.

## Declaration of Generative AI and AI-Assisted Technologies in the Writing Process

During the preparation of this work, the authors used ChatGPT to improve readability and language. After using this tool, the authors reviewed and edited the content as needed and take full responsibility for the content of the final article.

## CRediT Author Statement

**Salem Al-Jundi:** Conceptualization, Writing – original draft, Software, Writing – review and editing, Visualization. **Reshmi Augustine:** Data Curation, Writing – review and editing, Investigation, Validation.

## Data Availability

Mendeley DataS. Al-Jundi, Dataset on tax evasion in Baghdad, Iraq, Mendeley Data V1 (2025). https://doi.org/10.17632/G75TVDVRZG.1. (Original data).

## Supplementary Materials

The questionnaire and supplementary data for this article can be found online at https://data.mendeley.com/datasets/g75tvdvrzg/1.
